# The Role of Interleukin-4 and Interleukin-10 in Osteoarthritic Joint Disease: A Systematic Narrative Review

**DOI:** 10.1177/19476035221098167

**Published:** 2022-05-12

**Authors:** E.M. van Helvoort, E. van der Heijden, J.A.G. van Roon, N. Eijkelkamp, S.C. Mastbergen

**Affiliations:** 1 Department of Rheumatology & Clinical Immunology, UMC Utrecht, Utrecht University, Utrecht, The Netherlands; 2 Center of Translational Immunology, UMC Utrecht, Utrecht University, Utrecht, The Netherlands

**Keywords:** interleukins, osteoarthritis, osteoarthritis disease modification

## Abstract

**Objective:**

A fusion protein of interleukin-4 and interleukin-10 (IL4-10 FP) was developed as a disease-modifying osteoarthritis drug (DMOAD), and chondroprotection, anti-inflammation, and analgesia have been suggested. To better understand the mechanisms behind its potential as DMOAD, this systematic narrative review aims to assess the potential of IL-4, IL-10 and the combination of IL-4 and IL-10 for the treatment of osteoarthritis. It describes the chondroprotective, anti-inflammatory, and analgesic effects of IL-4, IL-10, and IL4-10 FP.

**Design:**

PubMed and Embase were searched for publications that were published from 1990 until May 21, 2021 (moment of search). Key search terms were: Osteoarthritis, Interleukin-4, and Interleukin-10. This yielded 2,479 hits, of which 43 were included in this review.

**Results:**

IL-4 and IL-10 showed mainly protective effects on osteoarthritic cartilage *in vitro* and *in vivo*, as did IL4-10 FP. Both cytokines showed anti-inflammatory effects, but also proinflammatory effects. Only *in vitro* IL4-10 FP showed purely anti-inflammatory effects, indicating that proinflammatory effects of one cytokine can be counteracted by the other when given as a combination. Only a few studies investigated the analgesic effects of IL-4, IL-10 or IL4-10 FP. *In vitro*, IL-4 and IL4-10 FP were able to decrease pain mediators. *In vivo*, IL-4, IL-10, and IL4-10 FP were able to reduce pain.

**Conclusions:**

In conclusion, this review describes overlapping, but also different modes of action for the DMOAD effects of IL-4 and IL-10, giving an explanation for the synergistic effects found when applied as combination, as is the case for IL4-10 FP.

## Introduction

Osteoarthritis (OA) is a progressive joint disease characterized by changes in multiple joint tissues, leading to pain, stiffness, and loss of function.^1^ Cartilage, bone, and synovial tissue show prominent structural changes in OA, and pain is the most important symptom and the reason for patients with OA to seek medical assistance. An ideal OA treatment not only reduces symptoms but also prevents further structural damage by combining chondroprotective, anti-inflammatory, and analgesic effects all in 1 disease-modifying OA drug (DMOAD). None of the current potential DMOADs have yet been approved for the treatment of OA by regulatory authorities worldwide. As criteria for approval of a DMOAD, a drug needs to provide both structural and clinical improvement.^2,3^ The persisting effort over the past years into development of new DMOADs has generated promising leads and candidate therapies. One of these promising approaches is the usage of anabolic stimuli.

**Figure 1.**
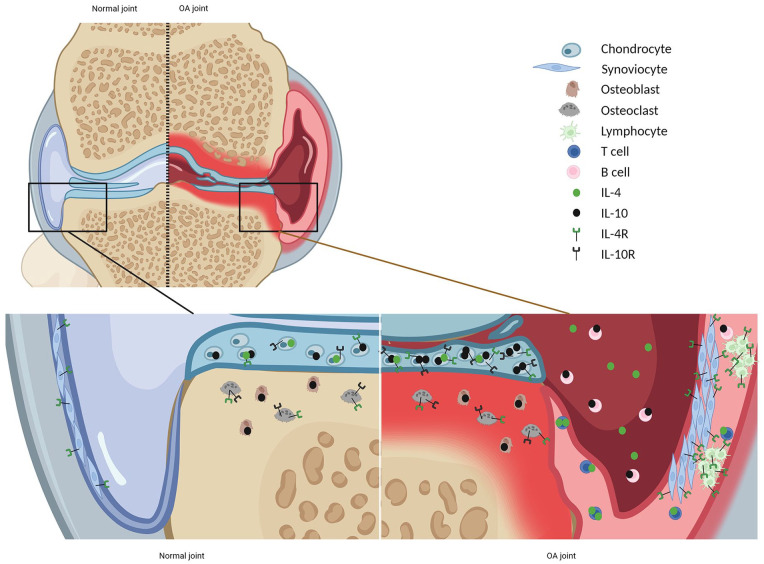
Expression of IL-4, IL-10, and their receptors (IL-4R and IL-10R) in the healthy and osteoarthritic joint. Created by Biorender.com. In healthy joints, IL-4, IL-10, and their receptors IL-4R and IL-10R are found in multiple joint tissues.^4,7-9^ In OA, chondrocytes express more IL-10 and receptors for IL-10 and IL-4 (IL10R, IL4R).^6,7^ In contrast, the expression of IL-4 is less in OA cartilage compared with normal cartilage.^10^ The OA synovium is infiltrated by multiple inflammatory cells, increasing the expression of IL-4 (e.g., produced by Th2 cells)^5^ and IL-10 (e.g., produced by B-cells),^11^ and IL-4R is found in lymphocyte aggregates.^9^ IL-4 = interleukin-4; IL-10 = interleukin-10; IL-4R = IL-4 receptor; IL-10R = IL-10 receptor; OA = osteoarthritis.

Regulatory cytokines such as interleukin (IL)-4 and IL-10, well known for their anti-inflammatory activity, are also anabolic stimulating cytokines that are produced by a variety of immune cells. IL-4 acts via 2 types of heterodimeric IL-4 receptors (IL-4R), expressed on numerous cell types, both immune and nonimmune cells. Type 1 consists of the IL-4Rα subunit and the common γ chain (γc), whereas type 2 consists of IL-4Rα and IL-13Rα1 chains.^4,5^ The IL-10 receptor is composed of 2 subunits, IL-10R1 and IL-10R2,^5^ and is also expressed on immune and nonimmune cells. In the osteoarthritic joint, increased expression of both IL-4 and IL-10 receptors has been demonstrated. For example chondrocytes express the IL-10R^6,7^ and both types of IL-4R.^4,7^ An overview of the expression of IL-4, IL-10, and their receptors (IL-4R and IL-10R) in the healthy and osteoarthritic joint is provided in **Figure 1**.

Importantly, IL-4 and IL-10 have chondroprotective effects. *In vivo*, IL-4 influences proteoglycan metabolism by inhibition of matrix metalloproteinases (MMPs) and prevents apoptosis of chondrocytes and fibroblast-like synoviocytes.^5^ IL-10 stimulates the synthesis of collagen type II and aggrecan, 2 important proteins in the extracellular matrix (ECM) of cartilage; affects proteoglycan metabolism; reduces MMPs; and, like IL-4, prevents apoptosis of chondrocytes.^5^

IL-4 and IL-10 have been tested for their anti-inflammatory and chondroprotective effects for the treatment of rheumatoid arthritis (RA); however, results were inconsistent. IL-10 has some proinflammatory properties, including stimulation of B-cell activity and upregulation of Fc receptors on antigen-presenting cells that in certain conditions could counteract its strong anti-inflammatory properties. This along with the short half-life of IL-10 has been suggested as potential explanations for the somewhat disappointing results. Both *in vitro* and *in vivo*, IL-10 was shown to increase Fc receptors on myeloid cells in the circulation of RA patients treated with IL-10.^12^ Nonetheless, in experimental *in vitro* and *in vivo* models, IL-10 (and IL-4) strongly prevented inflammation-induced cartilage degeneration, with combining both cytokines having additive effects. IL-10 also directly stimulated proteoglycan synthesis in cartilage explants.^13^ Moreover, in psoriatic arthritis, significant immune modulation was found after subcutaneously administered IL-10; however, no beneficial effects were found on clinical manifestations.^14^

Recently, the interest in IL-4 and IL-10 as therapeutics has been fueled by the development of a fusion protein of IL-4 and IL-10 (IL4-10 FP) to promote efficacy of the individual cytokines by facilitating synergy, promoting unique signaling^15^ and enhanced bioavailability.^16^

Since its development, the effects of IL4-10 FP have been evaluated in multiple studies and joint diseases. IL4-10 FP reversed persistent inflammatory pain in multiple mouse models,^16^ protected against blood-induced cartilage damage and inhibited production of proinflammatory cytokines *in vitro*,^17,18^ attenuated cartilage damage but not synovial inflammation in a mice model of hemophilic arthropathy,^17^ and reduced disease severity in established experimental arthritis in mice.^18^

Compared with healthy cartilage, OA cartilage expresses increased levels of IL-4R and IL-10R,^7^ indicating OA cartilage may become more responsive for the effects of IL4-10 FP. Studies evaluating the efficacy of IL4-10 FP in OA indeed show promising results. IL4-10 FP has chondroprotective and anti-inflammatory effects in OA cartilage explants^7^ and chondroprotective and analgesic effects in a canine OA model, as well as in a rat OA model.^19,20^

This systematic narrative review aims to assess the potential of IL-4, IL-10, and the combination of IL-4 and IL-10 for the treatment of OA. It describes the chondroprotective, anti-inflammatory, and analgesic effects of IL-4, IL-10, and IL4-10 FP to better understand the mechanisms behind its potential as a DMOAD.

## Methods

### Study Selection

PubMed and Embase were searched for publications that were published from 1990 until May 21, 2021 (moment of search). Key search terms were: Osteoarthritis, Interleukin-4, and Interleukin-10 (for full search strategies, see supplementary file 1). Duplicates were removed and the remaining articles were screened based on title and abstract by 2 reviewers (E.H. and E.M.H.). Disagreements between reviewers were resolved by consensus including a third reviewer (S.C.M.). Eligibility criteria were English language and availability of full text. All study types, describing direct effects of IL-4, IL-10 or IL4-10 FP on OA joint tissues or patient-reported outcome measures, were included. Reviews were excluded, but their reference lists were checked for additional articles.

### Data Collection

Full texts of included articles were screened by 2 authors (E.H. and E.M.H.) to extract information on (1) cytokine of interest, (2) experimental setup, and (3) chondroprotective, anti-inflammatory, and/or analgesic effects described. Remaining articles were grouped and described per DMOAD characteristic (chondroprotective, anti-inflammatory, and analgesic).

## Results

**Figure 2.**
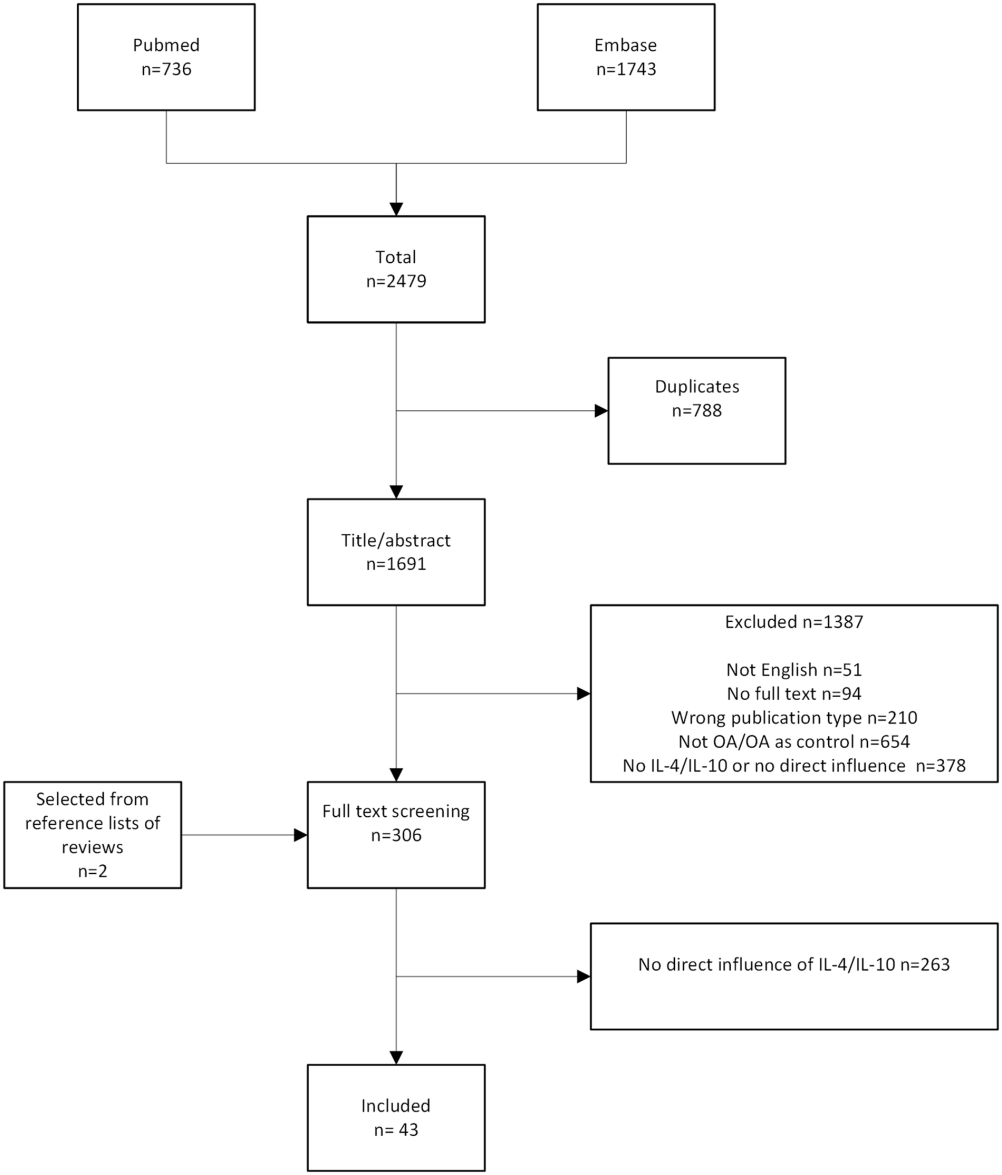
Selection process. OA = osteoarthritis; IL-4 = interleukin-4; IL-10 = interleukin-10.

**Figure 3.**
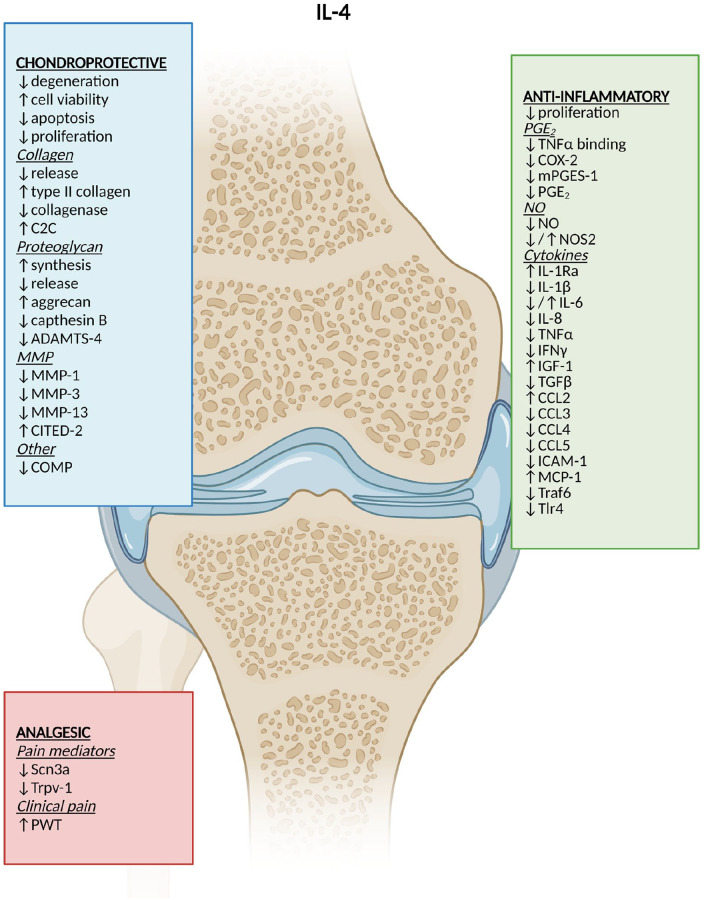
DMOAD effects of IL-4. Created by Biorender.com. ↓ = reduction; ↑ = increase; ↓/↑ = different results; C2C = collagen type II C-terminal cleavage neoepitope; DMOAD = disease-modifying osteoarthritis drug; ADAMTS = A desintegrin and metalloproteinase with thrombospondin motifs; MMP = matrix metalloproteinase; CITED-2 = Cbp/P300 interacting transactivator with Glu/Asp-rich carboxy terminal domain 2; COMP = cartilage oligomeric matrix protein; PGE_2_ = prostaglandin E_2_; TNFα = tumor necrosis factor-alpha; COX-2 = cyclooxygenase-2; mPGES-1 = microsomal prostaglandin E synthase-1; NO = nitric oxide; NOS2 = nitric oxide synthase-2; IL = interleukin; IFNγ = interferon gamma; IGF-1 = insulin growth factor-1; TGFβ = tumor growth factor-beta; CCL = chemokine (C-C motif) ligand; ICAM-1 = intercellular adhesion molecule-1; MCP-1 = monocyte chemoattractant protein-1; Scn3a = sodium voltage–gated channel alpha subunit 3 coding gene; Trpv1 = transient receptor potential cation channel subfamily V member 1 coding gene; PWT = paw-withdrawal threshold.

**Figure 4.**
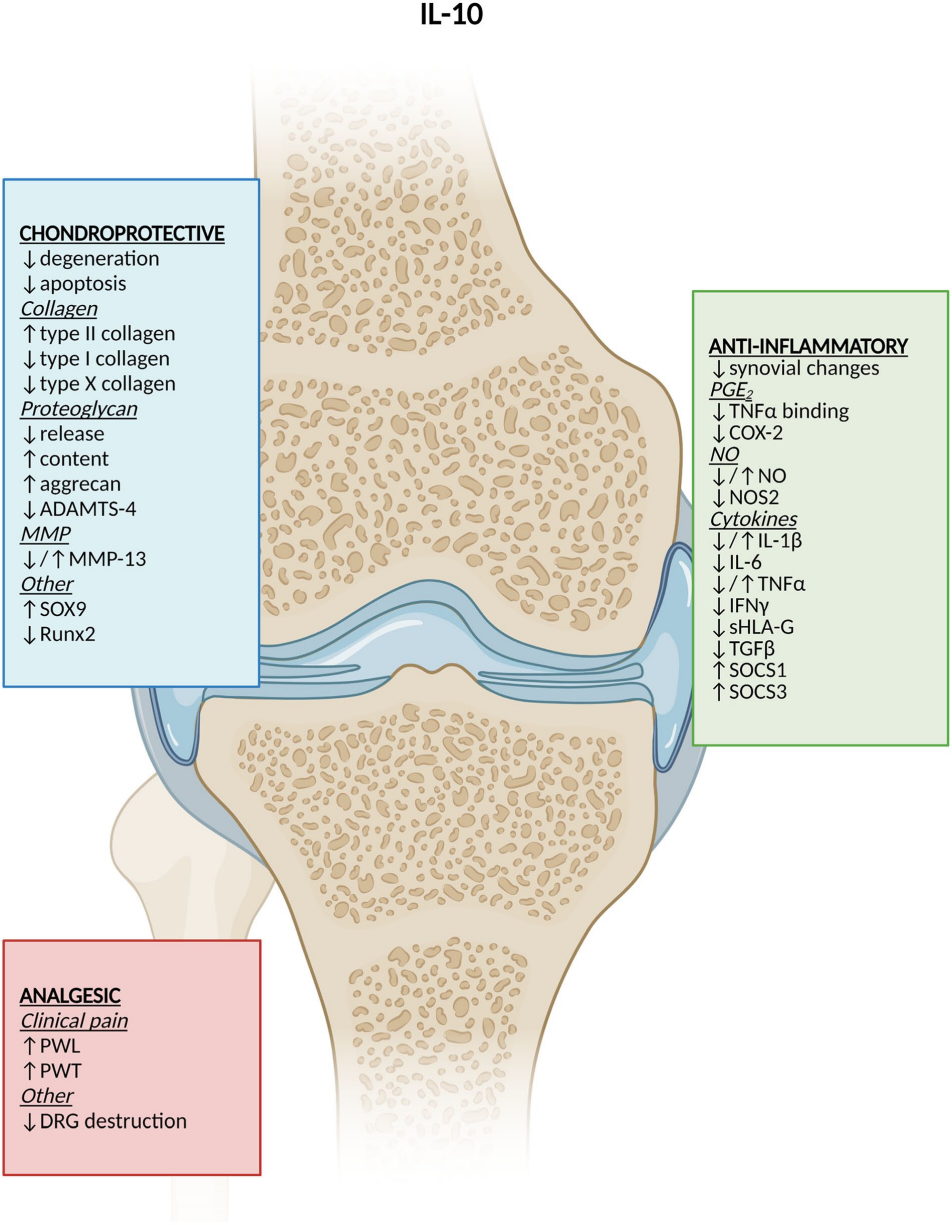
DMOAD effects of IL-10. Created by Biorender.com. ↓ = reduction; ↑ = increase; ↓/↑ = different results; ADAMTS = A desintegrin and metalloproteinase with thrombospondin motifs; DMOAD = disease-modifying osteoarthritis drug; MMP = matrix metalloproteinase; SOX9 = transcription factor sox 9 coding gene; PGE_2_ = prostaglandin E_2_; TNFα = tumor necrosis factor-alpha; COX-2 = cyclooxygenase-2; NO = nitric oxide; NOS2 = nitric oxide synthase-2; IL = interleukin; IFNγ = interferon gamma; (s)HLA-G = (soluble) human leukocyte antigen G; TGFβ = tumor growth factor-beta; SOCS = suppressor of cytokine signaling; PWL = paw-withdrawal latency; PWT = paw-withdrawal threshold; DRG = dorsal root ganglia.

**Figure 5.**
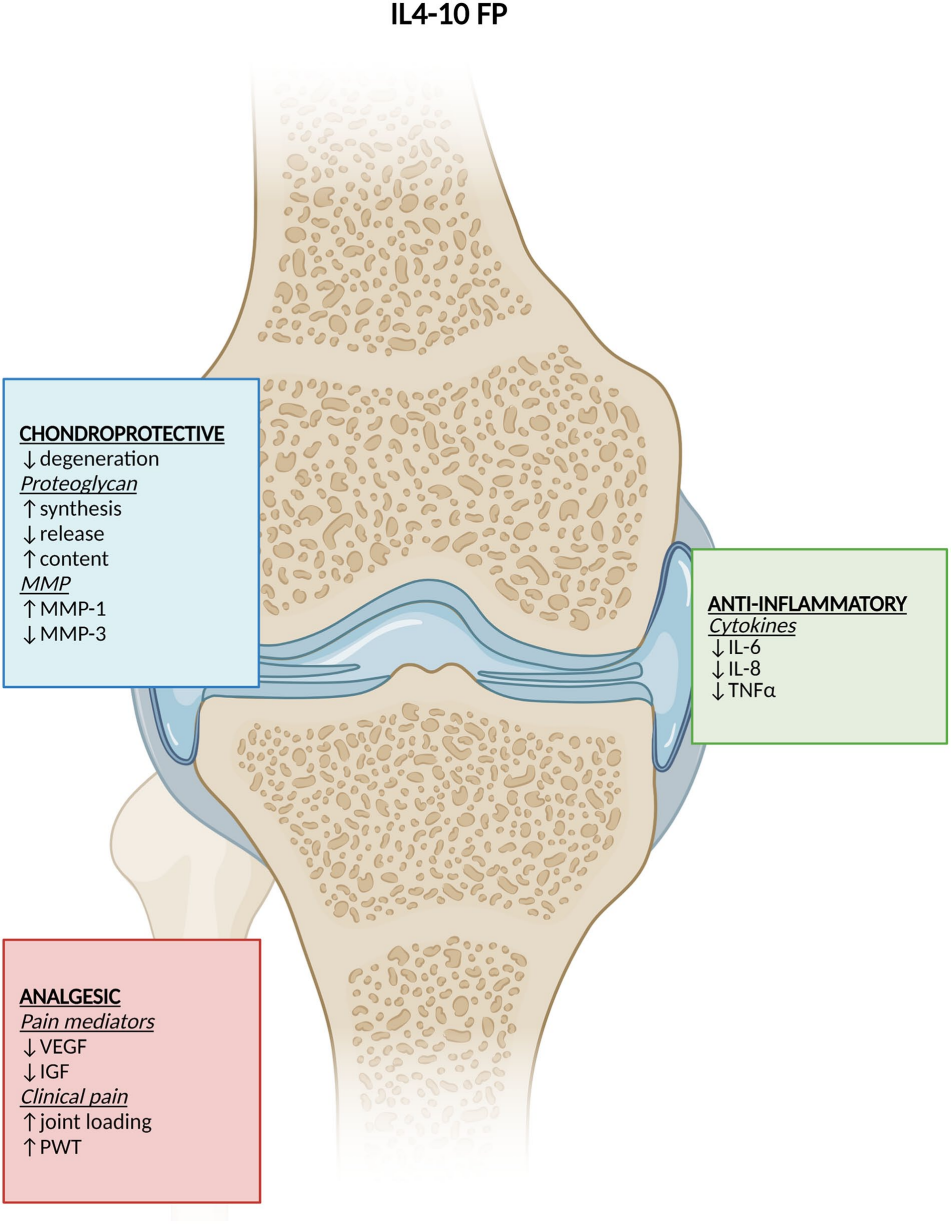
DMOAD effects of IL4-10 fusion protein. Created by Biorender.com. ↓ = reduction; ↑ = increase; ↓/↑ = different results; DMOAD = disease-modifying osteoarthritis drug; MMP = matrix metalloproteinase; IL = interleukin; TNFα = tumor necrosis factor-alpha; VEGF = vascular endothelial growth factor; IGF = insulin growth factor; PWT = paw-withdrawal threshold.

The initial search yielded 2,479 results. After removal of duplicates (*n* = 788), 1,691 articles remained. Selection on title/abstract led to an additional 1,387 exclusions for several reasons: not written in English (*n* = 51), no full text available (*n* = 94), wrong publication type (e.g., review, letter to the editor, commentary on original article, *n* = 210), not about OA or OA only used as control group (*n* = 654), and IL-4, IL-10, or IL4-10 FP not used as intervention (only as outcome measurement) (*n* = 378). Reference lists of excluded reviews fetched two more articles eligible for inclusion, which led to a total of 306 articles for screening of full text. Of the 306 articles, 263 did not describe an effect of IL-4 or IL-10 on OA joint tissues or patient-reported outcome measures and were excluded. The remaining 43 articles were included in this review (**Fig. 2**). See supplementary file 2 for an overview of included articles and their main findings (also in **Figs. 3-5**).

### Chondroprotective Effects

A total of 26 articles investigated the chondroprotective effects of either IL-4 or IL-10 or a combination of both.

In unstimulated human OA cartilage explants, IL-4 had no effect on glycosaminoglycan (GAG) release or on expression of enzymes that can induce GAG release (A desintegrin and metalloproteinase with thrombospondin motifs [ADAMTS] 4 and 5).^21^ Similarly, macrophage-conditioned medium (MCM) of human monocyte–derived macrophages stimulated with IL-4 (M(IL-4)) did not affect GAG release and expression of ADAMTS-4 and ADAMTS-5, COL2A1, MMP-1, or MMP-13.^22^

In chondrocytes stimulated with inflammatory cytokines, IL-4 treatment had positive effects on collagen levels. IL-4 reduced collagenase production,^8,23^ increased the messenger RNA (mRNA) expression of collagen type II,^24-26^ and lowered the release of total collagen.^25^ This was confirmed by immunohistochemistry.^24^ In addition, IL-4 increased the expression of proteoglycans^24^ by enhancing synthesis^27^ and reducing release.^25^ More specifically, IL-4 normalized the IL-1β and tumor necrosis factor-alpha (TNFα)-induced reduction of aggrecan mRNA expression,^24,25^ reduced mRNA expression of cathepsin B after cyclic tensile stress,^28^ and reduced ADAMTS-4 mRNA expression, but without effect on protein expression.^10^ IL-4 also reduced the synthesis of MMPs at mRNA and protein levels,^8,24,26,10,29-32^ and induced CBP/P300-interacting transactivator 2 (CITED2), which downregulates MMP-13.^31^ Knockdown of IL-4 in IL-1β-stimulated chondrocytes decreased cell viability and increased apoptosis,^33^ indicating an essential role of IL-4 in regulating survival of chondrocytes. IL-4 reduced IL-1β-induced proliferation of dedifferentiated chondrocytes, but not primary chondrocytes.^8,27^

In human OA cartilage explants that were exposed to mechanical compression, IL-4 protected the explants against histological degeneration and it increased the number of transcription factor SOX9-expressing chondrocytes compared with uncompressed cartilage explants. IL-4 did not affect SOX9 mRNA expression.^21^ This protective effect of IL-4 was confirmed in rat chondrocytes exposed to cyclic tensile stress, where IL-4 decreased the MMP-13 synthesis after mechanical loading.^28^ Furthermore, neutralizing IL-4 antibodies blocked the positive effects of mechanical stimulation (increased aggrecan and decreased MMP-13) in normal chondrocytes but not in OA chondrocytes, suggesting an important role of IL-4 in the anabolic response to mechanical stimulation in healthy cartilage, but not in diseased cartilage.^34^

In an *in vivo* rat model for OA, induced by anterior cruciate ligament tear and medical meniscectomy, IL-4-transfected spheroids of mesenchymal stem cells (MSCs) reduced chondrocyte apoptosis, signs of histological cartilage degeneration, and MMP-13 expression in cartilage tissue.^26^ In an IL-4 knockout mice model exposed to treadmill running, CITED2 mRNA and protein levels in cartilage tissue were lower compared with wild-type mice, while MMP-13 levels were slightly higher,^31^ confirming that CITED2 is a pivotal downstream molecule in IL-4-mediated MMP-13 reduction. Finally, intra-articular injection with IL-4 inhibited cartilage destruction in 2 surgically induced OA models.^31,35^

In conclusion, IL-4 showed no chondroprotective effects in unstimulated OA cartilage explants. However, IL-4 has chondroprotective effects on cytokine-stimulated chondrocytes, mechanically stimulated human cartilage explants, and multiple *in vivo* animal models.

In unstimulated chondrocytes, IL-10 did not affect MMP-13 (which cleaves collagen type II and was measured by collagenase 3) levels,^23^ but an adenoviral vector overexpressing IL-10 slightly increased MMP-13 expression.^36^ In addition, in unstimulated human OA cartilage explants, M(IL-10) MCM had no effect on COL2A1 expression.^22^ In line with this, overexpression of IL-10 in bone marrow mesenchymal stem cells (BM-MSCs) with Adeno-Associated Virus (AAV) IL-10 did not affect GAG release or content.^32^

In contrast, BM-MSCs reduced MMP-13 expression in IL-1β/TNFα-stimulated cartilage explants, but BM-MSCs transduced with AAV null had the same effect, suggesting that the positive effect is not related to IL-10 expression.^32^ Nevertheless, in isolated chondrocytes stimulated with inflammatory cytokines, IL-10 overexpression (using an adenovirus or lentivirus, respectively) upregulated collagen type II and aggrecan,^36,37^ and downregulated collagen type X^37^ mRNA and MMP-13 expression.^36^ Recombinant (r)IL-10 also impaired MMP-13 expression, but had no effect on TNFα-mediated aggrecan expression.^36^

IL-10 reduced injury-induced chondrocyte apoptosis, COL10A1 and COL1A1 expression, GAG release, MMP-13 synthesis, and ADAMTS-4 mRNA expression.^38,39^ In addition, IL-10 restored COL2A1 expression and increased GAG content and mRNA expression of aggrecan and transcription factor SOX9.^39^

*In vivo*, in a murine collagenase-induced OA model, human MSCs overexpressing viral IL-10, a product of Epstein-Barr virus that exhibits 84% amino acid sequence homology with human IL-10, reduced the percentage of cluster of differentiation (CD)4+ and CD8+ T-cells in popliteal lymph nodes; however, histologically, no effects on cartilage were found.^40^ In a rabbit model, the combination of IL-10 and IL-1Ra gene therapy markedly reduced cartilage pathology and decreased proteoglycan loss, and had greater chondroprotective effects than either of these cytokines alone.^41^

These data indicate that like IL-4, IL-10 had no effect in unstimulated chondrocytes and explants, with one study reporting an increase in MMP-13 expression after treatment with an adenoviral vector overexpressing IL-10. However, IL-10 had chondroprotective effects in cytokine-stimulated chondrocytes and cartilage explants, and apoptosis of injury-induced chondrocytes.

Studies in which the fusion protein of both cytokines (IL4-10 FP) was used revealed that IL4-10 FP normalized the OARSI cartilage structural damage score that was increased in the canine or rat OA Groove model.^19^ Moreover, IL4-10 FP increased proteoglycan synthesis and reduced proteoglycan release in OA cartilage explants,^7,19^ and normalized the reduced proteoglycan content in an *in vivo* canine OA model.^19,20^ IL4-10 FP reduced MMP-3 expression, but slightly increased MMP-1 expression in OA chondrocytes, whereas in synovial fibroblasts both were reduced. The release of tissue inhibitor of metalloproteinases (TIMP-1, which inhibits MMPs) was not affected by IL4-10 FP.^7^

### Anti-inflammatory Effects

Even more articles (*n* = 35) reported on anti-inflammatory effects of both cytokines in OA models on multiple levels.

IL-4 may exert anti-inflammatory action because it is able to compromise the binding of TNFα to its cell-surface receptors in synovial fibroblasts. IL-4 mildly upregulated cell-surface TNFR, but in addition increases the level of soluble TNFR-75, competing with cell-surface TNFR for binding of TNFα.^42^ IL-4 increased IL-6^43^ and chemokine (C-C motif) ligand (CCL2) production^44^ in OA synovial fibroblasts, indicating some proinflammatory effects in OA tissue. However, IL-4 also antagonized interferon gamma (IFNγ)-induced expression of intercellular adhesion molecule 1^43^ and promoted the expression of the anti-inflammatory cytokine IL-1Ra.^44^ Besides, IL-4 inhibited expression of cyclooxygenase 2 (COX-2), the main enzyme for prostaglandin (PGE) E_2_ production after TNFα stimulation,^42^ and inhibited IL-1β-induced proliferation^8^ and PGE_2_ production^44^ of synoviocytes. IL-4 reduced IL-1β and TNFα production in lipopolysaccharide (LPS)-stimulated^45^ or leukotriene B_4_ (LTB_4_)-stimulated OA synovium,^46^ but had no effect on these cytokines in unstimulated tissue.^46^

Next to cells from the synovial tissue, chondrocytes are able to produce inflammatory mediators as well. In unstimulated human OA chondrocytes, IL-4 had no effect on levels of arachidonic acid, phospholipase A2 (PLA2), COX-2, microsomal PGE synthase (mPGES)-1, or PGE_2_ expression,^47-49^ suggesting a PLA2 and eicosanoid-independent mechanism of action.^48^ Indeed, addition of the nitric oxide synthesis (NOS) inhibitor ι-NIO abolished the effects of IL-4, suggesting an NO-dependent mechanism of IL-4 in the downregulation of IL-1β-induced PGE_2_,^27^ but no direct effect of IL-4 on NO production was found in primary human OA chondrocytes.^8^

IL-4 inhibits NO production in chondrocytes stimulated with proinflammatory cytokines,^8,24-27,30,50^ although no effect was found on the IL-17-induced NO production in human OA chondrocytes.^51^ IL-4 inhibited CCL5, CCL3, and CCL4 mRNA and protein expression, but did not affect C-X-C motif chemokine (CXCL) 1 and CXCL8 expression.^10^ Knockdown of IL-4 led to an increase in TNFα, IFNγ, and IL-6 in IL-1β-stimulated chondrocytes.^33^

Pretreatment of rat chondrocytes with IL-4 reduced mechanical stress–induced expression of IL-1β.^28^ Similarly, rIL-4 suppressed the mechanical stress–induced iNOS mRNA and NO expression in rat chondrocytes.^35^

Transfection of IL-1β/TNFα-stimulated canine chondrocytes with IL-4 gene therapy using a COX-2 or cytomegalovirus (CMV) promotor reduced COX-2 and mPGES-1 expression, and with that downregulated PGE_2_ release^25,30^ and reduced the production of IL-1β, TNFα, IL-6, and IL-8.^24,25,29,30^ Furthermore, IL-4 upregulated IL-1Ra^25,30^ and insulin-like growth factor 1 (IGF-1; responsible for *de novo* synthesis of collagen and upregulation of proteoglycan production)^25,30^ and reduced the expression of binding protein and receptors for IGF-1.^24^ Besides, neutralizing IL-4 prevented downregulation of NO.^25^

Intra-articular injection with rIL-4 in a rat OA model decreased the population of NT-positive chondrocytes (a measurement for NO mediated tissue damage).^35^ Intra-articular administered IL-4 MSC spheroids reduced the expression of the inflammatory mediators Traf6 and Tlr4.^26^

Altogether, in the majority of studies, IL-4 had anti-inflammatory effects on cartilage and synovium, yet some proinflammatory effects (e.g., increased IL-6 and CCL2 production) in synovial fibroblasts were also reported. In unstimulated chondrocytes, IL-4 did not have any detectable effects, while in stimulated chondrocytes (either by cytokines or by mechanical stimulation) and *in vivo* models, IL4 had anti-inflammatory effects.

Two studies evaluated the effects of IL-10 on histological synovitis. One study reported no effect of IL-10 gene therapy on histological synovitis in a rabbit OA model,^41^ and another study reported less synovial changes in a murine collagenase-induced OA model after injection with humans MSCs overexpressing vIL-10.^40^

Like IL-4, IL-10 is also able to compromise the binding of TNFα to its cell-surface receptors in synovial fibroblasts. IL-10 increases the level of sTNFR-75 and reduces the expression of cell-surface TNFR.^42^ In synovial fibroblasts, IL-10 increased the expression of the anti-inflammatory human leukocyte antigen G^52^ and inhibited the expression of COX-2.^42^ In contrast, IL-10 had an opposing stimulatory effect on the expression of proinflammatory cytokines IL-1β and TNFα in LPS-stimulated^45^ or LTB_4_-stimulated OA synovium.^46^ In 3-dimensional synovial micromasses generated from primary synovial cells from OA patients who were stimulated with LPS, TNFα, or IL-1β, rIL-10 induced suppressor of cytokine signaling (SOCS) 3 and reduced LPS-induced IL-1β and TNFα expression.^53^ In synovial fluid, IL-10 suppressed proliferation and IFNγ expression of autologous T-cells.^11^

In unstimulated OA cartilage samples, M(IL-10) MCM increased IL-1β and SOCS1.^22^ Similarly, in cartilage explants stimulated with IFNγ and TNFα, to simulate inflammation, M(IL-10) MCM increased NO production.^22^ The BM-MSCs overexpressing IL-10 (using adenovirus or lentivirus) reduced IL-1β and IL-6 expression in IL-1β/TNFα-stimulated cartilage explants.^32^ Again, BM-MSCs transfected with AAV null had the same effects, suggesting that the anti-inflammatory effect is also not mediated by IL-10.^32^ Indeed, overexpression of IL-10 using an adenoviral vector did not influence IL-1β or IL-6 production, but did, however, increase TNFα production.^36^ Similar to IL-4, IL-10 had also no effect on levels of arachidonic acid, PLA2, COX-2, mPGES-1, or PGE_2_ expression in unstimulated human OA chondrocytes.^47-49^

In contrast to unstimulated chondrocytes, overexpression of IL-10 reduced IL-1β and IL-6 expression in IL-1β-stimulated chondrocytes.^36,37^ IL-10 had no effect on IL-1β^50^ or IL-17-induced NO production in chondrocytes.^51^

In bovine cartilage explants, IL-10 did not affect basal NO expression, but it did reverse the increase in NO and NOS2 mRNA expression after mechanical injury.^38^

IL-10 gene therapy using a CXCL10 promoter reduced IL-1β and IL-6 expression in the previously mentioned synovial micromasses.^53^

Overall, IL-10 has some proinflammatory effects in synovial fibroblasts and cartilage samples, but anti-inflammatory effects when chondrocytes were stimulated.

The fusion protein of IL-4 and IL-10 reduced the release of IL-6 and IL-8 by OA synovial tissue and cartilage explants *in vitro*,^7^ and canine IL4-10 FP reduced TNFα production in LPS-stimulated canine whole blood cultures.^19^ In *in vivo* OA models, these effects were less clear, mainly due to the usage of noninflammatory OA models.^19,20^

### Analgesic Effects

Only 3 studies reported on the effects of IL-4 and/or IL-10 on pain mediators. IL-4 could not restore vascular endothelial growth factor (VEGF) expression in human cartilage explants,^21^ which was increased after compression. In a rat OA model, intra-articular implantation of IL-4 MSC spheroids reduced the expression of Scn3a and TRPV1, 2 pain-related ion channels, in the spinal cord.^26^ *In vitro*, IL4-10 FP inhibited the release of VEGF and nerve growth factor, 2 pain-related mediators, from OA synovial tissue. In OA cartilage, only VEGF was significantly inhibited.^7^

In an *in vivo* rat model for OA induced by anterior cruciate ligament tear and medical meniscectomy, IL-4 MSC spheroids decreased mechanical allodynia.^26^ IL-10 knockout (IL-10^−/−^) mice develop stronger signs of pain such as thermal hyperalgesia and mechanical allodynia after chemically induced OA (monoiodoacetate model [MIA]) compared with control mice. Moreover, dorsal root ganglia were destructed in MIA-injected IL-10^−/−^ mice, while normal morphology was maintained in MIA-injected control mice. These results suggest that IL-10 deficiency exacerbated pain progression.^54^ In companion dogs with naturally occurring OA intra-articular injection with IL-10, encoding plasmid DNA decreased pain as measured by a visual analogue scale.^55^

In animal models, joint loading is often used as a surrogate for pain, where more joint loading indicates less joint pain. In the canine Groove model, intra-articular injections with IL4-10 FP led to increased joint loading (i.e., less pain), which lasted for approximately 1 day.^7,19^ In the rat Groove model, IL4-10 FP had a transient analgesic.^20^

In summary, IL-4 has analgesic effects *in vivo* but likely not through VEGF, as VEGF expression in human cartilage explants was unaffected. IL-10 and IL4-10 FP reduced OA-associated pain behaviors *in vivo.* IL4-10 FP reduced the expression of 2 pain mediators (VEGF and NGF) *in vitro*.

## Discussion

This systematic narrative review describes the chondroprotective, anti-inflammatory, and analgesic effects, the three pillars of a successful DMOAD, of the anti-inflammatory cytokines IL-4 and IL-10, and the IL4-10 FP. In general, both cytokines show promising effects on all 3 outcomes, as did the IL4-10 FP. Regarding chondroprotection, multiple studies describe a lack of effect of the separate cytokines, and 1 study reported negative effects of rIL-10 (increased MMP-13).^36^ In addition, some studies reported proinflammatory effects, despite IL-4 and IL-10 being anti-inflammatory cytokines. However, the IL4-10 FP showed purely anti-inflammatory effects, suggesting that by combining both cytokines into one treatment, the anti-inflammatory effects of one cytokine can counteract the possible proinflammatory effects of the other, and vice versa. It was shown previously that by using the IL4-10 FP, indeed the adverse effects of IL-10 are prevented by IL-4.^18^ Also, in blood-induced cartilage damage, the combination of IL-4 and IL-10 has advantages over IL-10 monotherapy,^56,57^ confirming the beneficial effects of combining both cytokines.

Next to the advantage of counteracting each other’s possible adverse effects, the IL4-10 FP also provides the possibility of synergy between IL-4 and IL-10. Both cytokines act via a different intracellular pathway, leading to additive anti-inflammatory effects.^57,58^ Indeed, the IL4-10 FP was more effective in treating persistent inflammatory hyperalgesia in an *in vivo* mice model, compared with IL-4 or IL-10, as well as to the combination of both separate cytokines.^16^ Moreover, IL4-10 FP inhibited inflammatory mediator-induced neuronal sensitization more effectively than the combination of both separate cytokines.^15^ Mechanistically, IL4-10 FP clustered IL-4Ra and IL-10Ra, whereas the combination of cytokines did not. This unique receptor clustering caused activation of an signaling cascade that strongly differed from that induced by the combination of cytokines, possibly explaining the superior effects of the IL4-10 FP.^15^ Moreover, by combining both cytokines into one molecule, the molecular weight increases, leading to improved bioavailability. Sialylation of the IL4-10 FP increased the molecular weight even further and resulted in higher half-life compared with the half-life of both cytokines alone.^59^

The Food and Drug Administration (FDA) and European Medicines Agency (EMA) require a DMOAD to slow down joint space narrowing on x-rays and relieve clinical symptoms.^2,3^ Despite many attempts, leading to promising results in phase II and III trials,^60^ there is still no approved DMOAD available. TissueGene-C (a mixture of human allogeneic chondrocytes and irradiated cells engineered to overexpress transforming growth factor-β1) showed chondroprotective and analgesic effects in a rat OA model, accompanied by increased IL-10 expression, suggestive for anti-inflammatory effects.^61^ In humans, intra-articular injection with TissueGene-C led to less structural progression.^62^ In addition, in a phase III trial, symptomatic benefits were found.^63^ A noninferiority trial comparing diacerein (inhibitor of IL-1β) and celecoxib (a selective COX-2 inhibitor) showed that diacerein was noninferior to celecoxib regarding symptomatic benefit and showed a good safety profile.^64^ A phase III trial evaluating the effects of tocilizumab in hand OA showed similar pain relief in the tocilizumab group compared with the placebo group.^65^

Multiple fusion proteins are developed in the search for a DMOAD as well. The OSCAR-Fc protein (fusion between osteoclast-associated receptor and the Fc part of human immunoglobulin 1) reduced cartilage damage in 2 *in vivo* mice models,^66^ but other pillars of a DMOAD therapy still need to be investigated. A fusion protein of tumor growth factor-β and latency-associated peptide (LAP-MMP-mTGF-β3) showed promising results in a rat OA model.^67^ This LAP has also been used for TIMP-3.^68^ These fusion proteins are developed to reduce side effects of the active component, rather than combining 2 active components (they are biologically inactive until cleavage of the LAP to release the therapy). This also accounts for the HB-NC4 (heparin binding domain-N-terminal non-collagenous domain 4) fusion protein, which had been developed to overcome the main limitation of NC4 therapy, namely, targeting ability.^69^ In contrast, the IL4-10 FP is the only fusion protein designed to combine the effects of 2 active signaling components.

The search for a successful DMOAD is continuing, and the Wnt/β-catenin signaling pathway inhibitor Lorecivivint, the bisphosphonate zoledronic acid, and multiple anti-inflammatory agents successful in the treatment of RA are tested in phase III trials in OA patients,^60^ and for Vitamin D, a phase IV trial is being conducted in OA.^60^ For now, the IL4-10 FP is one of the few compounds which has shown promising results on all 3 aspects of ideal DMOAD therapy in *in vitro* and *in vivo* animal models, although not in 1 model. Still, a lot of work is needed to develop the IL4-10 FP as a DMOAD, suitable for clinical practice. At present, in OA, the IL4-10 FP is envisioned to be used as intra-articular treatment, to reduce the possibility of systemic side effects,^19,20^ and with that is not usable for OA in smaller joints or in patients with polyarthritis. A major drawback of direct intra-articular application as applied so far is the rapid clearance out of the joint cavity due to their relatively low molecular weight, leading to only relative short transient effects and the necessity of weekly injections. More sustained effects on one injection, or other delivery routes need to become key in future studies to ensure clinical application.

At the same time, the concept of OA being a heterogeneous disease existing of multiple phenotypes is getting more and more attention, and future studies are needed to decide which patients should form the ideal target population for IL4-10 FP treatment (but also for other possible DMOADs). The canine and rat Groove model of OA mimic early post-traumatic OA, and inflammation was too mild to evaluate anti-inflammatory effects.^19,20^ The IL4-10 FP consists of 2 anti-inflammatory cytokines, and in that line of thought, it seems reasonable to assume that patients with an important inflammatory component as underlying mechanism for OA will most likely benefit from IL4-10 FP treatment.

## Supplemental Material

sj-docx-1-car-10.1177_19476035221098167 – Supplemental material for The Role of Interleukin-4 and Interleukin-10 in Osteoarthritic Joint Disease: A Systematic Narrative Review Click here for additional data file. Supplemental material, sj-docx-1-car-10.1177_19476035221098167 for The Role of Interleukin-4 and Interleukin-10 in Osteoarthritic Joint Disease: A Systematic Narrative Review by E.M. van Helvoort, E. van der Heijden, J.A.G. van Roon, N. Eijkelkamp and S.C. Mastbergen in CARTILAGE

sj-docx-2-car-10.1177_19476035221098167 – Supplemental material for The Role of Interleukin-4 and Interleukin-10 in Osteoarthritic Joint Disease: A Systematic Narrative Review Click here for additional data file. Supplemental material, sj-docx-2-car-10.1177_19476035221098167 for The Role of Interleukin-4 and Interleukin-10 in Osteoarthritic Joint Disease: A Systematic Narrative Review by E.M. van Helvoort, E. van der Heijden, J.A.G. van Roon, N. Eijkelkamp and S.C. Mastbergen in CARTILAGE
