# Supplementary material for: The Role of Interleukin-4 and Interleukin-10 in Osteoarthritic Joint Disease: A Systematic Narrative Review
Source: Cartilage. 2022 May 12;13(2):19476035221098167. doi: 10.1177/19476035221098167 (PMC9251827; doi:10.1177/19476035221098167)
Supplement: sj-docx-1-car-10.1177_19476035221098167 – Supplemental material for The Role of Interleukin-4 and Interleukin-10 in Osteoarthritic Joint Disease: A Systematic Narrative Review [file sj-docx-1-car-10.1177_19476035221098167.docx]

**Supplementary file 1: Search strategy for PubMed and Embase**

*Search strategy used for PubMed*

(interleukin-4[MeSH] OR interleukin 4[tiab] OR interleukin-4[tiab] OR interleukin4[tiab] OR il-4[tiab] OR il 4[tiab] OR il4[tiab] OR b-cell growth factor-1[tiab] OR b-cell growth factor 1[tiab] OR b cell growth factor 1[tiab] OR b cell growth factor-1[tiab] OR b-cell growth factor-i[tiab] OR b-cell growth factor i[tiab] OR b cell growth factor i[tiab] OR b cell growth factor-i[tiab] OR b-cell proliferating factor[tiab] OR b cell proliferating factor[tiab] OR b-cell stimulating factor-1[tiab] OR b-cell stimulating factor 1[tiab] OR b cell stimulating factor 1[tiab] OR b cell stimulating factor-1[tiab] OR b-cell stimulatory factor-1[tiab] OR b cell stimulatory factor 1[tiab] OR b-cell stimulatory factor 1[tiab] OR b cell stimulatory factor-1[tiab] OR bcgf-1[tiab] OR bcgf 1[tiab] OR bcgf1[tiab] OR binetrakin[tiab] OR bsf-1[tiab] OR bsf 1[tiab] OR bsf1[tiab] OR mast cell growth factor-2[tiab] OR mast cell growth factor 2[tiab] OR mcgf-2[tiab] OR mcgf 2[tiab] OR mcgf2[tiab] OR b lymphocyte stimulating factor 1[tiab] OR b lymphocyte stimulating factor-1[tiab] OR b-lymphocyte stimulating factor 1[tiab] OR b-lymphocyte stimulating factor-1[tiab] OR interleukin-10[MeSH] OR interleukin-10[tiab] OR interleukin 10[tiab] OR interleukin10[tiab] OR il-10[tiab] OR il 10[tiab] OR il10[tiab] OR cytokine synthesis inhibitory factor[tiab] OR csif[tiab] OR csif-10[tiab] OR csif 10[tiab] OR csif10[tiab] OR regulatory cytokine*[tiab] OR anti-inflammatory cytokine*[tiab] OR antiinflammatory cytokine*[tiab] OR anti inflammatory cytokine*[tiab]) AND (osteoarthritis[MeSH] OR osteoarthr*[tiab] OR osteo arthr*[tiab] OR osteo-arthr*[tiab] OR arthros*[tiab] OR oa[tiab] OR degenerative arthr*[tiab] OR osteoarthrosis deformans[tiab] OR noninflammatory arthrosis[tiab] OR degenerative joint disease[tiab] OR osteoarthritis cartilage[jour])

| Search | Query | Items found |
| --- | --- | --- |
| #13 | #8 AND #12 | 649 |
| #12 | #9 OR #10 OR #11 | 136956 |
| #11 | osteoarthritis cartilage[jour] | 4242 |
| #10 | osteoarthr*[tiab] OR osteo arthr*[tiab] OR osteo-arthr*[tiab] OR arthros*[tiab] OR oa[tiab] OR degenerative arthr*[tiab] OR osteoarthrosis deformans[tiab] OR noninflammatory arthrosis[tiab] OR degenerative joint disease[tiab] | 119408 |
| #9 | osteoarthritis[Mesh] | 61926 |
| #8 | #3 OR #6 OR #7 | 105867 |
| #7 | regulatory cytokine*[tiab] OR anti-inflammatory cytokine*[tiab] OR antiinflammatory cytokine*[tiab] OR anti inflammatory cytokine*[tiab] | 12492 |
| #6 | #4 OR #5 | 64827 |
| #5 | interleukin-10[tiab] OR interleukin 10[tiab] OR interleukin10[tiab] OR il-10[tiab] OR il 10[tiab] OR il10[tiab] OR cytokine synthesis inhibitory factor[tiab] OR csif[tiab] OR csif-10[tiab] OR csif 10[tiab] OR csif10[tiab] | 62331 |
| #4 | interleukin-10[MeSH] | 27119 |
| #3 | #1 OR #2 | 52429 |
| #2 | interleukin 4[tiab] OR interleukin-4[tiab] OR interleukin4[tiab] OR il-4[tiab] OR il 4[tiab] OR il4[tiab] OR b-cell growth factor-1[tiab] OR b-cell growth factor 1[tiab] OR b cell growth factor 1[tiab] OR b cell growth factor-1[tiab] OR b-cell growth factor-i[tiab] OR b-cell growth factor i[tiab] OR b cell growth factor i[tiab] OR b cell growth factor-i[tiab] OR b-cell proliferating factor[tiab] OR b cell proliferating factor[tiab] OR b-cell stimulating factor-1[tiab] OR b-cell stimulating factor 1[tiab] OR b cell stimulating factor 1[tiab] OR b cell stimulating factor-1[tiab] OR b-cell stimulatory factor-1[tiab] OR b cell stimulatory factor 1[tiab] OR b-cell stimulatory factor 1[tiab] OR b cell stimulatory factor-1[tiab] OR bcgf-1[tiab] OR bcgf 1[tiab] OR bcgf1[tiab] OR binetrakin[tiab] OR bsf-1[tiab] OR bsf 1[tiab] OR bsf1[tiab] OR mast cell growth factor-2[tiab] OR mast cell growth factor 2[tiab] OR mcgf-2[tiab] OR mcgf 2[tiab] OR mcgf2[tiab] OR b lymphocyte stimulating factor 1[tiab] OR b lymphocyte stimulating factor-1[tiab] OR b-lymphocyte stimulating factor 1[tiab] OR b-lymphocyte stimulating factor-1[tiab] | 49138 |
| #1 | interleukin-4[MeSH] | 22609 |

*Search strategy used for Embase:*

('interleukin 4'/exp OR 'interleukin 4':ti,ab,kw OR 'interleukin-4':ti,ab,kw OR 'interleukin4':ti,ab,kw OR 'il-4':ti,ab,kw OR 'il 4':ti,ab,kw OR 'il4':ti,ab,kw OR 'b-cell growth factor-1':ti,ab,kw OR 'b-cell growth factor 1':ti,ab,kw OR 'b cell growth factor 1':ti,ab,kw OR 'b cell growth factor-1':ti,ab,kw OR 'b-cell growth factor-i':ti,ab,kw OR 'b-cell growth factor i':ti,ab,kw OR 'b cell growth factor i':ti,ab,kw OR 'b cell growth factor-i':ti,ab,kw OR 'b-cell proliferating factor':ti,ab,kw OR 'b cell proliferating factor':ti,ab,kw OR 'b-cell stimulating factor-1':ti,ab,kw OR 'b-cell stimulating factor 1':ti,ab,kw OR 'b cell stimulating factor 1':ti,ab,kw OR 'b cell stimulating factor-1':ti,ab,kw OR 'b-cell stimulatory factor-1':ti,ab,kw OR 'b-cell stimulatory factor 1':ti,ab,kw OR 'b cell stimulatory factor 1':ti,ab,kw OR 'b cell stimulatory factor-1':ti,ab,kw OR 'bcgf-1':ti,ab,kw OR 'bcgf 1':ti,ab,kw OR 'bcgf1':ti,ab,kw OR 'binetrakin':ti,ab,kw OR 'bsf-1':ti,ab,kw OR 'bsf 1':ti,ab,kw OR 'bsf1':ti,ab,kw OR 'mast cell growth factor-2':ti,ab,kw OR 'mast cell growth factor 2':ti,ab,kw OR 'mcgf-2':ti,ab,kw OR 'mcgf 2':ti,ab,kw OR 'mcgf2':ti,ab,kw OR 'b lymphocyte stimulating factor 1':ti,ab,kw OR 'b lymphocyte stimulating factor-1':ti,ab,kw OR 'b-lymphocyte stimulating factor 1':ti,ab,kw OR 'b-lymphocyte stimulating factor-1':ti,ab,kw OR ‘interleukin 10’/exp OR 'interleukin-10':ti,ab,kw OR 'interleukin 10':ti,ab,kw OR 'interleukin10':ti,ab,kw OR 'il-10':ti,ab,kw OR 'il 10':ti,ab,kw OR 'il10':ti,ab,kw OR 'cytokine synthesis inhibitory factor':ti,ab,kw OR 'csif':ti,ab,kw OR 'csif-10':ti,ab,kw OR 'csif 10':ti,ab,kw OR 'csif10':ti,ab,kw OR 'regulatory cytokine$':ti,ab,kw OR 'anti-inflammatory cytokine$':ti,ab,kw OR 'antiinflammatory cytokine$':ti,ab,kw OR 'anti inflammatory cytokine$':ti,ab,kw) AND (‘osteoarthritis’/exp OR 'osteoarthr*':ti,ab,kw OR 'osteo arthr*':ti,ab,kw OR 'osteo-arthr*':ti,ab,kw OR 'arthros*':ti,ab,kw OR 'oa':ti,ab,kw OR 'degenerative arthr*':ti,ab,kw OR 'osteoarthrosis deformans':ti,ab,kw OR 'noninflammatory arthrosis':ti,ab,kw OR 'degenerative joint disease':ti,ab,kw OR ‘osteoarthritis cartilage’:ta)

| No. | Query | Results |
| --- | --- | --- |
| #14 | #13 AND [embase]/lim | 1418 |
| #13 | #8 AND #12 | 1494 |
| #12 | #9 OR #10 OR #11 | 207291 |
| #11 | ‘osteoarthritis cartilage’:ta | 12237 |
| #10 | 'osteoarthr*':ti,ab,kw OR 'osteo arthr*':ti,ab,kw OR 'osteo-arthr*':ti,ab,kw OR 'arthros*':ti,ab,kw OR 'oa':ti,ab,kw OR 'degenerative arthr*':ti,ab,kw OR 'osteoarthrosis deformans':ti,ab,kw OR 'noninflammatory arthrosis':ti,ab,kw OR 'degenerative joint disease':ti,ab,kw | 164084 |
| #9 | ‘osteoarthritis’/exp | 129093 |
| #8 | #3 OR #6 OR #7 | 186761 |
| #7 | 'regulatory cytokine$':ti,ab,kw OR 'anti-inflammatory cytokine$':ti,ab,kw OR 'antiinflammatory cytokine$':ti,ab,kw OR 'anti inflammatory cytokine$':ti,ab,kw | 17919 |
| #6 | #4 OR #5 | 124315 |
| #5 | 'interleukin-10':ti,ab,kw OR 'interleukin 10':ti,ab,kw OR 'interleukin10':ti,ab,kw OR 'il-10':ti,ab,kw OR 'il 10':ti,ab,kw OR 'il10':ti,ab,kw OR 'cytokine synthesis inhibitory factor':ti,ab,kw OR 'csif':ti,ab,kw OR 'csif-10':ti,ab,kw OR 'csif 10':ti,ab,kw OR 'csif10':ti,ab,kw | 89504 |
| #4 | ‘interleukin 10’/exp | 113047 |
| #3 | #1 OR #2 | 91838 |
| #2 | 'interleukin 4':ti,ab,kw OR 'interleukin-4':ti,ab,kw OR 'interleukin4':ti,ab,kw OR 'il-4':ti,ab,kw OR 'il 4':ti,ab,kw OR 'il4':ti,ab,kw OR 'b-cell growth factor-1':ti,ab,kw OR 'b-cell growth factor 1':ti,ab,kw OR 'b cell growth factor 1':ti,ab,kw OR 'b cell growth factor-1':ti,ab,kw OR 'b-cell growth factor-i':ti,ab,kw OR 'b-cell growth factor i':ti,ab,kw OR 'b cell growth factor i':ti,ab,kw OR 'b cell growth factor-i':ti,ab,kw OR 'b-cell proliferating factor':ti,ab,kw OR 'b cell proliferating factor':ti,ab,kw OR 'b-cell stimulating factor-1':ti,ab,kw OR 'b-cell stimulating factor 1':ti,ab,kw OR 'b cell stimulating factor 1':ti,ab,kw OR 'b cell stimulating factor-1':ti,ab,kw OR 'b-cell stimulatory factor-1':ti,ab,kw OR 'b-cell stimulatory factor 1':ti,ab,kw OR 'b cell stimulatory factor 1':ti,ab,kw OR 'b cell stimulatory factor-1':ti,ab,kw OR 'bcgf-1':ti,ab,kw OR 'bcgf 1':ti,ab,kw OR 'bcgf1':ti,ab,kw OR 'binetrakin':ti,ab,kw OR 'bsf-1':ti,ab,kw OR 'bsf 1':ti,ab,kw OR 'bsf1':ti,ab,kw OR 'mast cell growth factor-2':ti,ab,kw OR 'mast cell growth factor 2':ti,ab,kw OR 'mcgf-2':ti,ab,kw OR 'mcgf 2':ti,ab,kw OR 'mcgf2':ti,ab,kw OR 'b lymphocyte stimulating factor 1':ti,ab,kw OR 'b lymphocyte stimulating factor-1':ti,ab,kw OR 'b-lymphocyte stimulating factor 1':ti,ab,kw OR 'b-lymphocyte stimulating factor-1':ti,ab,kw | 65994 |
| #1 | ‘interleukin 4’/exp | 79655 |
