# Supplementary material for: The Role of Interleukin-4 and Interleukin-10 in Osteoarthritic Joint Disease: A Systematic Narrative Review
Source: Cartilage. 2022 May 12;13(2):19476035221098167. doi: 10.1177/19476035221098167 (PMC9251827; doi:10.1177/19476035221098167)
Supplement: sj-docx-2-car-10.1177_19476035221098167 – Supplemental material for The Role of Interleukin-4 and Interleukin-10 in Osteoarthritic Joint Disease: A Systematic Narrative Review [file sj-docx-2-car-10.1177_19476035221098167.docx]

**Supplementary file 2**

**Table 1. Included articles**

| **Author** | **Publication year** | **Cytokine** | **Experimental set-up** | **Chondroprotective** | **Anti-inflammatory** | **Analgesic** |
| --- | --- | --- | --- | --- | --- | --- |
| Alaaeddine et al. | 1999 | IL-4 | TNFα-stimulated human OA synovial fibroblasts cultured with IL-4 |  | ↓ PGE_2_ |  |
|  |  | IL-10 | TNFα-stimulated human OA synovial fibroblasts cultured with IL-10 |  | ↓ PGE_2_ |  |
| Assirelli et al. | 2014 | IL-4 | IL-1β-stimulated human OA chondrocytes cultured with IL-4 | ↓ MMP-13  ↓ ADAMTS-4 | ↓ CCL3-5 |  |
| Behrendt et al. | 2016 | IL-10 | Bovine cartilage cultured with IL-10 before compression | ↓ apoptosis  ↓ proteoglycan release | ↓ NO |  |
| Behrendt et al. | 2018 | IL-10 | Bovine cartilage cultured with IL-10 after compression | ↓ apoptosis  ↑ proteoglycan content  ↓ aggrecan loss  ↓ hyaluronic acid  ↑ COL2A1  ↑ ACAN  ↑ SOX9  ↓ COL10A1  ↓ COL1A1 |  |  |
| Bendrups et al. | 1993 | IL-4 | LPS-stimulated human OA synovial tissue cultured with recombinant IL-4 |  | ↓ TNFα  ↓ IL-1β |  |
| Broeren et al. | 2016 | IL-10 | LPS/IL-1β/TNFα-stimulated 3D micromasses of human OA synovial tissue cultured with recombinant IL-10 |  | ↑ SOCS3  ↓ IL-1β  ↓ TNFα |  |
|  |  |  | LPS/IL-1β/TNFα-stimulated 3D micromasses of human OA synovial tissue transduced with IL-10 gene therapy using a CXCL10 vector |  | ↓ IL-1β  ↓ IL-6 |  |
| Cameron et al. | 2021 | IL-10 | IL-1β/TNFα-stimulated cartilage explants cultured with BM-MSCs overexpressing IL-10 using adenoviral vector | ↓ MMP-13 | ↓ IL-1β  ↓ IL-6  ↓ TNFα |  |
| Chowdhury et al. | 2006 | IL-4 | IL-1β-stimulated bovine chondrocyte/agarose constructs cultured with IL-4 w/wo compression | ↑ chondrocyte proliferation | ↓ NO  ↓ PGE_2_ |  |
| Doi et al. | 2008 | IL-4 | Rat chondrocytes cultured with IL-4 before cyclic tensile stress | ↓ MMP-13  ↓ Cathepsin B | ↓ IL-1β |  |
| Dolzani et al. | 2019 | IL-4 | IL-1β-stimulated human OA cartilage explants cultured with IL-4 before compression | ↓ OARSI histology score  ↓ COMP  ↑ C2C |  |  |
| Farrell et al. | 2016 | IL-10 | CIA mice model injected with hMSCs overexpressing vIL-10 using adenoviral vector |  | ↓ synovial changes |  |
| Fernandez et al. | 2003 | IL-10 | Human OA chondrocytes incubated with IL-10 |  | ↑ HO-1 |  |
| Fernandez et al. | 2004 | IL-10 | Human OA cartilage incubated with IL-10 |  | ↑ HO-1 |  |
|  |  |  | Human OA chondrocytes incubated with IL-10 |  | ↑ HO-1 |  |
| Guicheux et al. | 2002 | IL-4 | Human OA synoviocytes pre-treated with IL-4 before incubation with IL-1β | ↓ collagenase  ↓ MMP-13  ↓ proliferation  ↑ STAT6 phosphorylation |  |  |
|  |  |  | Primary human OA chondrocytes pre-treated with IL-4 before incubation with IL-1β | No effects on primary chondrocytes | | |
|  |  |  | Dedifferentiated human OA chondrocytes pre-treated with IL-4 before incubation with IL-1β | ↓ collagenase  ↓ MMP-13  ↓ proliferation  ↑ STAT6 phosphorylation | ↓ NO |  |
| He et al. | 2002 | IL-4 | LTB_4_-stimulated human OA synovial explants cultured with recombinant IL-4 |  | ↓ IL-1β  ↓ TNFα |  |
|  |  | IL-10 | LTB_4_-stimulated human OA synovial explants cultured with recombinant IL-10 |  | ↑ IL-1β  ↑ TNFα |  |
| He et al. | 2017 | IL-4 | IL-4 knockdown in IL-1β-stimulated human OA chondrocytes after knockdown of SOCS1 | ↓ viability  ↑ apoptosis  ↑ caspase 9 | ↑ TNFα  ↑ IFNγ  ↑ IL-6  ↑iNOS |  |
| He et al. | 2019 | IL-4 | Human OA chondrocytes treated with IL-4 and/or mechanical loading | ↑ CITED2  ↑ STAT6 phosphorylation  ↑ JAK3 phosphorylation  ↓ MMP-13 |  |  |
|  |  |  | IL-4 knockout mice subjected to treadmill running | ↓ CITED2  ↑ MMP-13 |  |  |
|  |  |  | Weekly i.a. injections with IL-4 in DMM mice model | ↓ OARSI score  ↑ CITED2  ↓ MMP-13 |  |  |
| Kojima et al. | 2004 | IL-4 | Human OA chondrocytes stimulated with IL-4 | No effects on mPGES-1 or COX2 mRNA expression | | |
|  |  | IL-10 | Human OA chondrocytes stimulated with IL-10 | No effects on mPGES-1 or COX2 mRNA expression | | |
| Kwon et al. | 2018 | IL-10 | IL-10 knockout MIA mice model | ↑ OARSI score |  | ↓ PWL |
| Lang et al. | 2014 | IL-4 | LPS-stimulated Equine chondrocytes transfected with IL-4 gene therapy using a COX2 promotor | ↓ MMP-1  ↓ MMP-3 | ↓ IL-1β  ↓ IL-8  ↓ IL-6 |  |
| Leistad et al. | 2011 | IL-4 | Human OA chondrocytes cultured with IL-4 |  | ↓ PGE_2_ |  |
| Manning et al. | 2010 | IL-4 | IL-1β/TNFα-stimulated canine chondrocytes transfected with IGF-1 w/wo IL-4 (co-expression increased expression of both cytokines) | ↓ MMP-1  ↓ MMP-3  ↓ MMP-13  ↑ aggrecan  ↑ type II collagen  ↑ proteoglycan content | ↓ IGFBP  ↓ IGFR1  ↓ IL-1β  ↓ TNFα  ↓ IL-6  ↓ iNOS  ↓ nitrite |  |
| Martel-Pelletier et al. | 1999 | IL-4 | IL-1β/TNFα/LIF-stimulated human OA chondrocytes cultured with IL-17 and IL-4 | No effects on IL-17 induced NO | | |
|  |  | IL-10 | IL-1β/TNFα/LIF-stimulated human OA chondrocytes cultured with IL-17 and IL-10 | No effects on IL-17 induced NO | | |
| Millward-Sadler et al. | 2000 | IL-4 | Human chondrocytes incubated with anti-IL-4 antibodies before mechanical stimulation | ↓ aggrecan  ↑ MMP-3 |  |  |
| Millward-Sadler et al. | 2006 | IL-4 | Human OA chondrocytes incubated with IL-4 | ↑ STAT6 phosphorylation |  |  |
| Müller et al. | 2008 | IL-10 | Unstimulated human chondrocytes treated with recombinant IL-10 |  | ↑ TNFα |  |
|  |  |  | TNFα-stimulated human chondrocytes treated with recombinant IL-10 |  | ↓ IL-1β |  |
|  |  |  | TNFα-stimulated human chondrocytes transduced with IL-10 using an adenoviral vector | ↑ aggrecan  ↓ MMP-13 |  |  |
| Ongaro et al. | 2010 | IL-10 | Human OA synovial fibroblasts |  | ↑ sHLA-G |  |
| Park et al. | 2020 | IL-4 | IL-1β/LPS-stimulated mice chondrocytes treated with IL-4 loaded microspheres |  | ↓ NO |  |
|  |  | IL-10 | IL-1β/LPS-stimulated mice chondrocytes treated with IL-10 loaded microspheres | No effects on NO production | | |
| Rachakonda et al. | 2008 | IL-4 | IL-1β/TNFα-stimulated canine chondrocytes transfected with IL-4 gene therapy using a COX2 promoter | ↓ MMP-3  ↓ MMP-13  ↓ TGFβ | ↓ IL-1β  ↓ IL-6  ↓ IL-8  ↓ TNFα  ↓ iNOS  ↓ COX2  ↓ mPGES-1  ↑ IGF-1  ↑ IL-1Ra  ↓ NO  ↓ PGE_2_ |  |
| Rai et al. | 2011 | IL-4 | IL-1β/TNFα-stimulated canine chondrocytes scaffolds transfected with IL-4 gene therapy using a COX2 promoter | ↑COL2A1  ↑ aggrecan  ↓ collagen release  ↓ proteoglycan release | ↓ IL-1β  ↓ IL-6  ↓ TNFα  ↓ iNOS  ↓ COX2  ↑ IGF-1  ↑ IL-1Ra  ↓ NO  ↓ PGE_2_ |  |
| Schlaak et al. | 1995 | IL-4 | Human OA synovial tissue incubated with IL-4 |  | ↑ IL-6  ↓ ICAM-1 |  |
|  |  | IL-10 | Human OA synovial tissue incubated with IL-10 | No effects on inflammatory cytokines | | |
| Seitz et al. | 1994 | IL-4 | IL-1β/TNFα-stimulated human OA synovial tissue pre-incubated with IL-4 |  | ↑ MCP-1  ↑ IL-1Ra  ↓ PGE |  |
| Song et al. | 2020 | IL-4 | IL-1β-stimulated rat chondrocytes incubated with IL-4 MSC (spheroids) | ↓ apoptosis  ↓ MMP-13  ↑ type II collagen | ↓ NO  ↓ iNOS |  |
|  |  | IL-4 | i.a. injection with IL-4 MSC (spheroids) in rat ACLT-MMx model | ↓ modified Mankin score  ↓ apoptosis  ↓ MMP-13 | ↓ NO | ↑ PWT  ↓ Scn3a  ↓ Trpv1  ↓ hypersensitivity spinal cord |
| Steen-Louws et al. | 2018 | IL4-10 FP | Human OA cartilage cultured with IL4-10 FP | ↑ proteoglycan synthesis  ↓ proteoglycan release  ↓ MMP-3 | ↓ IL-6  ↓ IL-8 |  |
|  |  |  | Human OA synovial tissue cultured with IL4-10 FP | ↓ MMP-1  ↓ MMP-3 | ↓ IL-6  ↓ IL-8 | ↓ VEGF  ↓ NGF |
|  |  |  | i.a. injection with IL4-10 FP in canine Groove model |  |  | ↓ joint loading |
| Sun et al. | 2019 | IL-10 | IL-10 producing B cells and T cells cultured with recombinant human IL-10R |  | ↑ T cell proliferation  ↑ IFNγ |  |
| Tardif et al. | 1999 | IL-4 | (IL-1β-stimulated) human OA chondrocytes incubated with IL-4 | ↓ collagenase 3 |  |  |
|  |  | IL-10 | Human OA chondrocytes incubated with IL-4 | No effects on collagenase 3 | | |
| Utomo et al. | 2016 | IL-4 | Human OA cartilage cultured with M(IL-4) MCM | No effects on gene expression | | |
|  |  |  | IFNγ/TNFα-stimulated human OA cartilage cultured with M(IL-4) MCM | ↓ ACAN |  |  |
|  |  | IL-10 | Human OA cartilage cultured with M(IL-4) MCM |  | ↑ SOCS1  ↑ IL-1β  ↑ NO |  |
|  |  |  | IFNγ/TNFα-stimulated human OA cartilage cultured with M(IL-10) MCM | No effects on gene expression | | |
| Van Helvoort et al. | 2019 | IL4-10 FP | LPS-stimulated canine whole blood cultured with IL4-10 FP |  | ↓ TNFα |  |
|  |  |  | TNFα-stimulated canine cartilage cultured with IL4-10 FP | ↑ proteoglycan synthesis |  |  |
|  |  |  | Weekly i.a. injections with IL4-10 FP in canine Groove model | ↓ OARSI score  ↑ proteoglycan content |  | ↑ joint loading |
| Van Helvoort et al. |  | IL4-10 FP | Weekly i.a. injections with IL4-10 FP in rat Groove model | ↓ OARSI score |  | ↓ PWT |
| Watkins et al. | 2020 | IL-10 | I.a. injection with IL-10 encoding plasmid DNA in dogs with naturally-occurring OA |  |  | ↓ pain |
| Yorimitsu et al. | 2008 | IL-4 | Rat chondrocytes pre-incubated with recombinant IL-4 before cyclic tensile stress |  | ↓ iNOS  ↓ NO |  |
|  |  |  | Daily i.a. injections with recombinant IL-4 in rat ACLT-MCLT model | ↓ cartilage destruction  ↓ proteoglycan loss | ↓ NO |  |
| Yu et al. | 2021 | IL-10 | IL-1β-stimulated human OA chondrocytes co-cultured with chondrocytes overexpressing IL-10 using a lentivirus vector | ↑ collagen type II  ↑ aggrecan  ↓ collagen type X  ↑ SOX9  ↓ Runx2 | ↓ IL-6 |  |
| Zhang et al. | 2004 | IL-10 | i.a. injection with (IL-1Ra and) IL-10 transfected rabbit synoviocytes in rabbit MCLT-MMx model | ↓ cartilage pathology  ↓ proteoglycan loss |  |  |

ACAN: Aggrecan coding gene, ACLT: Anterior cruciate ligament tear, ADAMTS: A desintegrin and metalloproteinase with thrombospondin motifs, BM-MSC: Bone marrow-derived Mesenchymal stem cell, C2C: Collagen type II C-terminal cleavage neoepitope, CCL: Chemokine (C-C motif) ligand, CIA: Collagen-induced arthritis, CITED2: Cbp/P300 Interacting transactivator with Glu/Asp rich carboxy terminal domain 2, COL2A1; Collagen type II alpha 1 chain coding gene, COMP: Cartilage oligomeric matrix protein, COX2: Cyclooxygenase-2, CXCL: Chemokine (C-X-C motif) ligand, DMM: Destabilization of the medical meniscus, FP: Fusion protein, (s)HLA-G: (soluble) Human leukocyte antigen G, HO-1: Heme oxygenase-1, i.a.: Intra-articular, ICAM: Intercellular adhesion molecule, IFN: Interferon, IGF: Insulin growth factor, IGFBP: IGF binding protein, IGFR: IGF receptor, IL: Interleukin, iNOS: Inducible nitric oxide synthase, JAK: Janus kinase, LPS: Lipopolysaccharide, LTB_4_: Leukotriene B4, MCLT: Medial collateral ligament tear, MCM: Macrophage conditioned medium, MCP: Monocyte chemoattractant protein, MIA: Monoiodoacetate-induced arthritis, MMP: Matrix metalloproteinase, MMx: medial meniscectomy, mPGES-1: Microsomal prostaglandin E synthase-1, mRNA: Messenger ribonucleic acid, (h)MSC: (human) Mesenchymal stem cell, NGF: nerve growth factor, NO: Nitric oxide, OA: Osteoarthritis, OARSI: Osteoarthritis Research Society International, PGE_2_: Prostaglandin E_2,_ PWL: Paw withdrawal latency, PWT: Paw withdrawal threshold, Scn3a: Sodium voltage-gated channel alpha subunit 3 coding gene, STAT: Signal transducer and activator of transcription, SOCS: Suppressor of cytokine signaling, SOX9: Transcription factor sox 9 coding gene, TNFα: Tumor necrosis factor alpha, Trpv1: Transient receptor potential cation channel subfamily V member 1 coding gene, VEGF: Vascular endothelial growth factor
